# Legal challenges and regulatory responses in online medical consultation

**DOI:** 10.3389/fdgth.2026.1957951

**Published:** 2026-10-26

**Authors:** Qi Chen

**Affiliations:** College of Applied Arts and Science, Beijing Union University, Beijing, China

**Keywords:** digital health, healthcare service, online medical consultation, legal challenges, regulatory responses, Beijing

## Abstract

Online medical consultation (OMC), a component of digital health, has expanded rapidly, giving rise to a variety of operational models. Furthermore, advances in artificial intelligence have created new opportunities for its evolution. With their abundant medical resources and technological capabilities, major cities such as Beijing have experienced particularly rapid growth in OMC. However, given the lack of OMC-specific regulations, the sector in Beijing still faces several legal uncertainties, notably regarding the legal status of OMC services and platform liability. Therefore, it is recommended that cities such as Beijing establish a dedicated OMC regulatory framework centered on comprehensive local departmental rules. Such rules can define the nature of services and platform liability under the overarching principle that standards should be tailored to the operational model.

## 1 Introduction

Digitalization has transformed many areas of society, including healthcare, leading to the rapid expansion of digital health services, including online medical consultation (OMC). These services have improved the accessibility of high-quality medical services, helping to overcome geographic and temporal constraints, and alleviating the uneven distribution of healthcare resources (1).

Once viewed as synonymous with telemedicine (2, 3), OMC is now recognized as one of its subtypes (4, 5). Telemedicine provides healthcare services via a variety of telecommunications technologies, including voice calls, text messaging, and radio communication (2). These services range from primary care and specialist referrals to remote patient monitoring, consumer health information, and medical education (6). Moreover, it can be used both between doctors and patients and among healthcare providers (6). In contrast, OMC refers to a specific health service delivered through digital platforms, where qualified healthcare practitioners register to provide consultation services to patients and health advice to the general public (7, 8).

OMC has expanded at a remarkable pace, growing by an average of 150% every 5 years (2, 9). The COVID-19 pandemic further accelerated its development, as it aligned with social distancing measures and effectively reduced cross-infection risks (10, 11). Following the pandemic, OMC has continued to evolve, as reflected in the increasing diversity of its operational models (Figure 1). Typically, users select registered healthcare practitioners on an OMC platform and interact via text, images, or audio calls (1). In this model, the platform provides medical consultation services and functions like an e-commerce platform, enabling communication between users and healthcare practitioners. Several new operational models have recently emerged. In one of these models, OMC services are delivered through internet hospitals, which are established independently by physical medical institutions, jointly by physical medical institutions and third-parties, or by enterprises relying on physical medical institutions. Another model involves instant messaging tools. OMC services are provided through a healthcare practitioner's personal account or dedicated practitioner-led chat groups.

**Figure 1:**
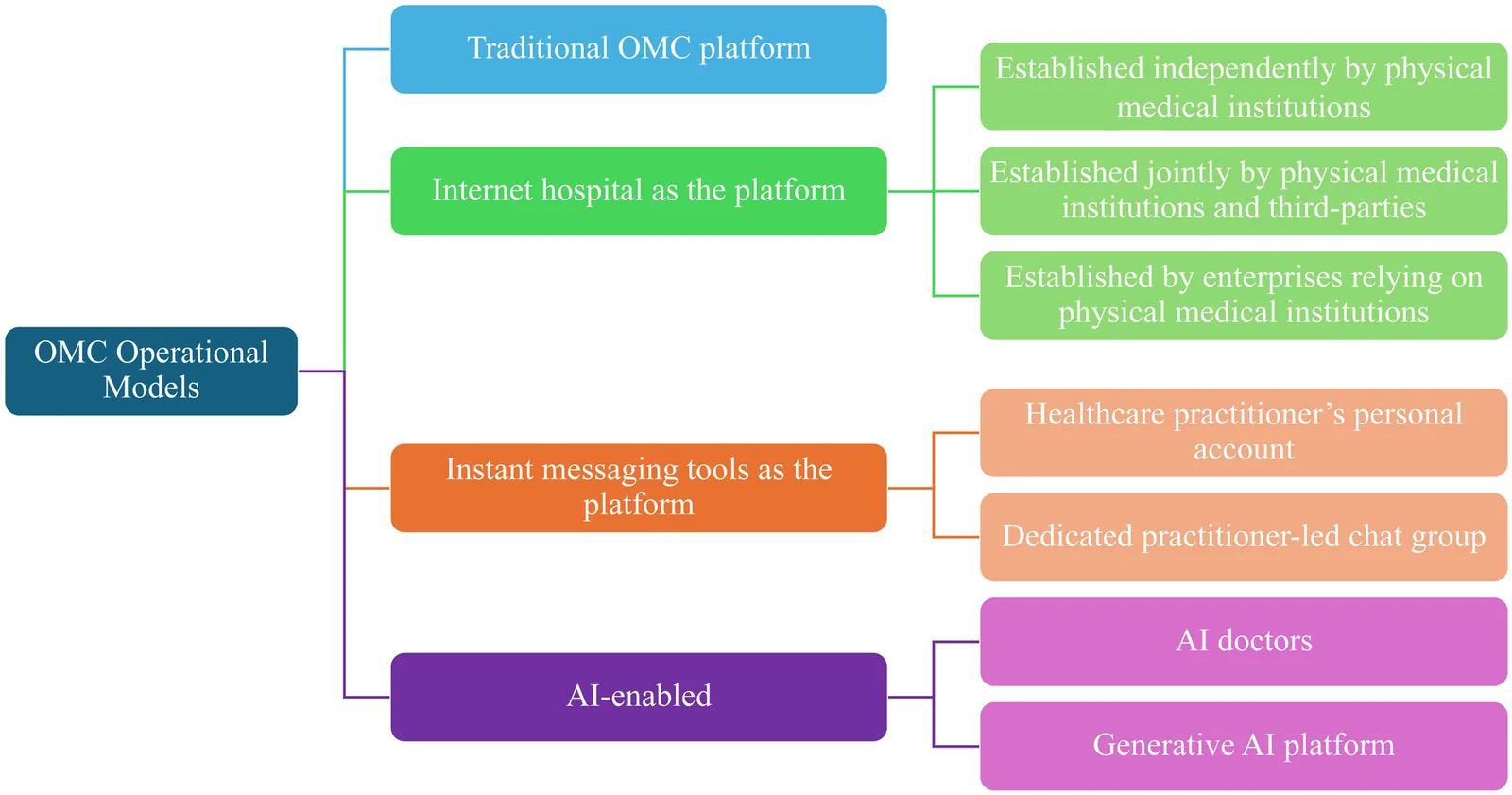
Operational models of online medical consultation (OMC).

Advances in artificial intelligence (AI) have introduced new dimensions to OMC. For example, healthcare practitioners are no longer the only providers of health advice; many platforms have introduced “AI doctors” that provide the interpretation of medical examination results, medication guidance, symptom assessment, and related services (12). Furthermore, users are no longer limited to OMC platforms and seek medical guidance directly through generative AI platforms, such as ChatGPT, DeepSeek, and Doubao (13, 14).

OMC has attracted substantial research interest, with studies focusing on patient usage and engagement (1, 11, 15), service satisfaction (16, 17), business models (7), and the patient–doctor relationship (18, 19). The rapid expansion of OMC has inevitably given rise to legal challenges, underscoring the urgent need for regulatory oversight.

## 2 Legal challenges of the OMC

The rapid development of OMC has largely been driven by an abundance of medical resources and technological innovations, leading to a broader applications and a larger user base in urban areas than in rural regions (15). Legal challenges arising from OMC are more likely to happen—and tend to be more complex—in urban settings. In this study, cases were retrieved from the Wolters Kluwer China Law & Reference database using the search terms “online medical consultation” and “internet healthcare” under two specific causes of action: “disputes over the right to life, bodily integrity, and health” and “disputes over medical malpractice liability”. The search was limited to cases decided from July 17,2018^1^ to the present, and returned 20 cases, 13 of which originated in Beijing. After further review, cases that merely mentioned the keywords were excluded, leaving 5 cases, all from Beijing. Accordingly, Beijing, a megacity, serves as the analytical context for examining the legal challenges associated with OMC application.

### 2.1 Development and regulations of the OMC in Beijing

Beijing has been at the forefront of OMC development in China and is the headquarters for several nationally recognized platforms, such as JD Health, WeDoctor, Haodf (Good Doctor Online), and Chunyu Doctor. During the COVID-19 pandemic, Beijing launched the Online Medical Consultation Platform for COVID-19, bringing together more than 1,000 doctors and 5 hospitals, all located in Beijing (20). Post-pandemic, Beijing continued to expand OMC services, and by July 2025, the city supported 102 internet hospitals and 307 medical institutions that provided online diagnosis and treatment services (21). The city also established a municipal-level unified platform enrolling 54 hospitals and nearly 9,000 doctors (21, 22). These digital facilities have seen active utilization, handling 1.12 million online consultations in the first 6 months of 2025, reflecting a 45% increase relative to the same period in 2024 (21).

The rapid growth of OMC in Beijing has attracted sustained regulatory attention (Table 1). At the national level, the primary regulations governing internet healthcare include the Measures for the Administration of Internet Diagnosis and Treatment (for Trial Implementation), the Measures for the Administration of Internet Hospitals (for Trial Implementation), and the Detailed Rules for the Supervision of Internet Diagnosis and Treatment (for Trial Implementation). Complementing these national measures, Beijing introduced local policies, such as the Beijing Special Plan for Medical and Health Facilities (2020–2035). This plan explicitly outlines a “1 + N + 1” internet healthcare framework consisting of one supervisory platform for internet diagnosis and treatment, multiple sub-platforms for diverse medical services, and one unified public service platform for internet hospitals. In accordance with this framework, the Beijing Municipal Health Commission established the Beijing Internet Diagnosis and Treatment Supervision Platform, mandating that all medical institutions delivering internet-based services connect to the platform and submit to regulatory oversight (23).

**Table 1:** Primary regulations and policies applicable to online medical consultations (OMC). .

Regulations and policies	Territorial scope	Effective date	Summary of relevant provisions
Measures for the Administration of Internet Diagnosis and Treatment (for Trial Implementation)	National	2018.07.17	Defines internet diagnosis and treatment, requiring that the service provider be a medical institution and that the services provided be limited to follow-up consultations. It permits cooperation with third-parties, provided that the responsibilities, rights and interests of all parties are clearly specified. It also imposes management and record-keeping responsibilities on medical institutions
Measures for the Administration of Internet Hospitals (for Trial Implementation)	National	2018.07.17	Defines the types of internet hospitals and requires that the services they provide be limited to follow-up consultations. It also imposes responsibilities for management, record-keeping, and data protection on internet hospitals. Additionally, it designates medical institutions as the liable entities and requires that, where medical institutions cooperate with third-parties, the responsibilities, rights and interests of all parties be clearly specified
Detailed Rules for the Supervision of Internet Diagnosis and Treatment (for Trial Implementation)	National	2022.02.08	Sets out detailed provisions on the responsibilities of medical institutions in internet diagnosis and treatment, covering areas such as management, personnel, and services
Notice of the Beijing Municipal Health Commission on Promoting the Application of the Beijing Internet Diagnosis and Treatment Supervision Platform	Beijing	2021.04.21	Establishes the Beijing Internet Diagnosis and Treatment Supervision Platform, mandating that all medical institutions providing Internet Diagnosis and Treatment connect to the platform and submit to its oversight
Beijing Special Plan for Medical and Health Facilities (2020–2035)	Beijing	2021.09.08	Envisions a “1 + N + 1” internet healthcare model that comprises one supervisory platform for internet diagnosis and treatment, multiple sub-platforms for diverse medical services, and one unified public service platform for internet hospitals

### 2.2 Legal challenges in the Beijing context

Although several regulations govern internet healthcare, they were primarily designed for internet diagnosis and treatment rather than OMC specifically. Furthermore, existing liability provisions focus on medical institutions rather than OMC platforms. Consequently, these frameworks do not address the unique legal issues arising from OMC applications. OMC, a component of digital health, integrated medical and digital domains, and AI integration adds a layer of complexity. This, combined with the regulatory gaps, creates recurring legal challenges, most notably regarding the legal characterization of OMC services and platform liability.

#### 2.2.1 The ambiguous nature of OMC services

When disputes arise from OMC usage, the initial challenge is classifying the nature of the service provided by OMC platforms. Specifically, it remains unclear whether these services constitute advisory services or as diagnosis and treatment. The Measures for the Administration of Internet Diagnosis and Treatment (for Trial Implementation) defines internet diagnosis and treatment as follow-up consultations for common and chronic diseases, as well as “Internet+” family doctor contract services, conducted by a medical institution using its own registered practitioners through internet and other informatic technology. The National Health Commission further explained that internet diagnosis and treatment occurs when practitioners reach a diagnostic conclusion about a disease and propose a treatment plan, such as prescribing medication or directing hospital treatment (24). In contrast, services involving no definitive diagnosis or treatment are categorized as advisory services (24). Consequently, service classification hinges on two factors: whether the provider is a medical institution whose practitioners are registered with that institution, and whether the service content involves definitive diagnosis and treatment.

Because traditional OMC platforms primarily connect users with healthcare practitioners and are not medical institutions themselves, the services they provide are generally classified as advisory services. This view is widely accepted in practice. For instance, platforms such as Chunyu Doctor explicitly state in user agreements that their recommendations rely solely on user descriptions and do not constitute a basis for diagnosis or treatment (25). Similarly, courts evaluating OMC disputers usually classify these interactions as advisory, a position endorsed by both the Beijing Chaoyang Primary People's Court and the Beijing Second Intermediate People's Court in their judgments (26, 27).

Nevertheless, real-world scenarios are often more complex. One case heard by the Beijing Third Intermediate People's Court involved a patient who, on the recommendation of the surgeon who had performed his operation, used the Chunyu Doctor platform for post-operative follow-up communication. While generally recognizing that OMC services are advisory, the court held that, in this case, the communication in question constituted medical diagnosis and treatment (28). This suggests that courts recognize that simply categorizing services provided by traditional OMC platforms as advisory is insufficient. Instead, the nature of such services must be determined according to the specific circumstances.

The increasingly diversified operational models of OMC add further complexity to this issue. As discussed earlier, OMC now encompasses a wide range of operational models provided across numerous platforms (Figure 1). The fact that a service provider is not a medical institution is no longer a sufficient basis for excluding its services from being classified as diagnosis and treatment. Moreover, a more precise standard for defining definitive diagnosis and treatment is needed to reflect current realities. Beijing, at the forefront of OMC development in China, hosts all these diverse models, making a clearer standard for defining the nature of OMC services urgently needed.

#### 2.2.2 Difficulties in determining OMC platform liability

Determining the liability of OMC platforms is another pivotal challenge when disputes arise from the use of their services. Although current regulations designate medical institutions as the primary liable entities, platforms are not entirely exempt from liability. In the absence of specific statutory provisions, OMC platforms are generally expected to meet fundamental regulatory obligations, including verifying the qualifications of registered practitioners and providing clear warnings that the services offered are advisory in nature and do not constitute medical diagnosis or treatment. This expectation rests on the premise that platforms function solely as intermediaries, with their provision classified as advisory services.

However, OMC platforms differ from ordinary online service platforms because their services directly affect user's fundamental rights to life and health. Merely verifying credentials and issuing disclaimers is therefore insufficient. As the Beijing Chaoyang Primary People's Court observed, while online platforms cannot be required to make precise medical judgements on all consulting information, they should nevertheless exercise prudent management, maintain retrievable records for future reference, and refrain from simply disclaiming all responsibility through a standard agreement disclaimers or brief reminders (26). The court regarded this duty as part of the social responsibility that such platforms ought to bear (26). Nevertheless, when and to what extent a platform should assume this social responsibility remains unresolved, and a uniform standard has yet to be established. Clarifying the circumstances under which the liability of a platform arises and defining the specific contours of this social responsibility are therefore essential.

The diversification of operational models also complicates the determination of platform liability. When a platform functions purely as an intermediary for medical advisory services, its liability remains largely confined to fundamental regulatory obligations. However, the proliferation of diverse operational structures raises a fundamental question regarding the platform's legal identity, namely, whether it acts merely as an intermediary, a healthcare provider, or a hybrid entity that combining elements of both.

The integration of AI introduces additional operational and legal dimensions. AI-based tools can generate inaccurate information through hallucinations or processing errors, raising critical legal questions. If a user suffers harm after relying on erroneous output from an AI tool—whether a generative AI application or an embedded AI assistant—the grounds, extent, and circumstances under which the platform should incur liability remain unestablished. Unresolved liability issues can hinder the continued growth of OMC, rendering clearer regulatory approaches essential.

## 3 Regulatory responses to the OMC

To address the legal challenges arising from the rapid development of OMC and thereby ensure its sustained growth, appropriate regulatory responses are needed. This is particularly important for megacities such as Beijing, which possess concentrated high-quality medical resources and advanced technological innovation capacity. Establishing well-designed regulatory responses in Beijing can promote the healthy and sustained development of OMC locally as well as serve as a model for other cities.

### 3.1 Establishing rules specifically tailored to the OMC

Well-designed regulatory responses requires rules tailored to OMC. Currently, the primary legal benchmarks are the Measures for the Administration of Internet Diagnosis and Treatment (for Trial Implementation), the Measures for the Administration of Internet Hospitals (for Trial Implementation), and the Detailed Rules for the Supervision of Internet Diagnosis and Treatment (for Trial Implementation). Courts rely on these instruments when adjudicating disputes. However, as discussed in Section 2.2, they were not designed for OMC and cannot address its distinct legal challenges, making a dedicated regulatory framework necessary.

The core of this framework should be a targeted administrative measure. Rather than high-level national legislation, this could take the form of local departmental rules jointly issued by municipal authorities, led by the municipal health commission—an initiative Beijing is well-positioned to spearhead. Unlike current regulations, which target medical institutions conducting Internet diagnosis and treatment, this instrument would focus on OMC and have OMC platforms as primary regulated entities. It would provide a clear definition of OMC based on three criteria: (1) health services delivered through digital platforms; (2) services provided by qualified healthcare practitioners registered on the platform; and (3) services intended exclusively for interactions between practitioners and patients or individuals seeking health advice. Beyond this definitional function, this instrument should be comprehensive in scope, cover key dimensions—including licensing, practice standards, oversight, and liability—to guide industry practice, administrative supervision, and judicial dispute resolution locally. In addition to providing legal grounding for local OMC activities, this instrument can also serve as a replicable model for other jurisdictions.

### 3.2 Classifying the nature of OMC services according to different operational models

To address the ambiguity regarding the legal nature of OMC services, this proposed departmental rule should set forth explicit classification provisions. Given current operational realities, this study recommends classifying OMC services according to their operational models (Table 2).

**Table 2:** Proposed regulatory framework for online medical consultation (OMC) based on operational models.

Operational model	Service nature		Liability allocation	Proposed platform responsibilities
Traditional OMC model	Initial consultation	Advisory services	The practitioner is the party liable for medical malpractice The platform bears fault-based liability	Verify practitioner credentials; warn users that services are advisory; maintain complete and retrievable records; assist vulnerable users
	Related follow-up within a reasonable timeframe after offline care	Diagnosis and treatment		
Internet hospital as the platform	Diagnosis and treatment		The medical institution is the party liable for medical malpractice The platform or AI developer bears fault-based liability	Fault-based liability under the cooperation agreement
Instant messaging tools as the platform	Initial consultation	Advisory services	The practitioner is the party liable for medical malpractice The platform bears fault-based liability	Maintain complete and retrievable records; assist vulnerable users
	Related follow-up within a reasonable timeframe after offline care	Diagnosis and treatment		
AI-enabled	Advisory services		The AI developer bears product liability The platform bears fault-based liability	Provide clear warnings that services are advisory; exercise prudent oversight; maintain complete and retrievable records; offer support to vulnerable users

Categorized regulation has been identified as an effective approach for managing rapid technological development, as reflected in several Chinese laws and regulations. For instance, the Data Security Law establishes a tiered data protection system, and the Provisional Measures for the Administration of Generative Artificial Intelligence Services adopts a categorized regulatory framework for generative AI. Legal scholarship similarly reflects this trend. Studies have proposed classifying AI systems before determining legal liability (29), implementing category-based data protection (30), and applying graded regulatory structure to healthcare AI applications (31). Given the diversity of OMC models, regulating services according to operational models provides a coherent regulatory pathway.

The central debate surrounding OMC services concerns whether they constitute advisory services or diagnosis and treatment. This study agrees with the statutory definition requiring diagnosis and treatment to involve disease diagnosis and treatment planning. However, the providers of such services should not be limited to medical institutions. Services provided by non-medical platforms should not be automatically excluded from classification as diagnosis and treatment, provided that they involve actual disease diagnosis and treatment planning.

This study proposes a four-step test (Figure 2) for distinguishing between advisory services and diagnosis and treatment. The first step involves determining whether the service is provided by a qualified practitioner. If it is not, the service should be classified as an advisory service. Services provided by AI are a primary example and should be classified as advisory because they do not involve human medical practitioners, and AI technology has neither advanced to nor been recognized as capable of independently performing diagnosis and treatment.

**Figure 2:**
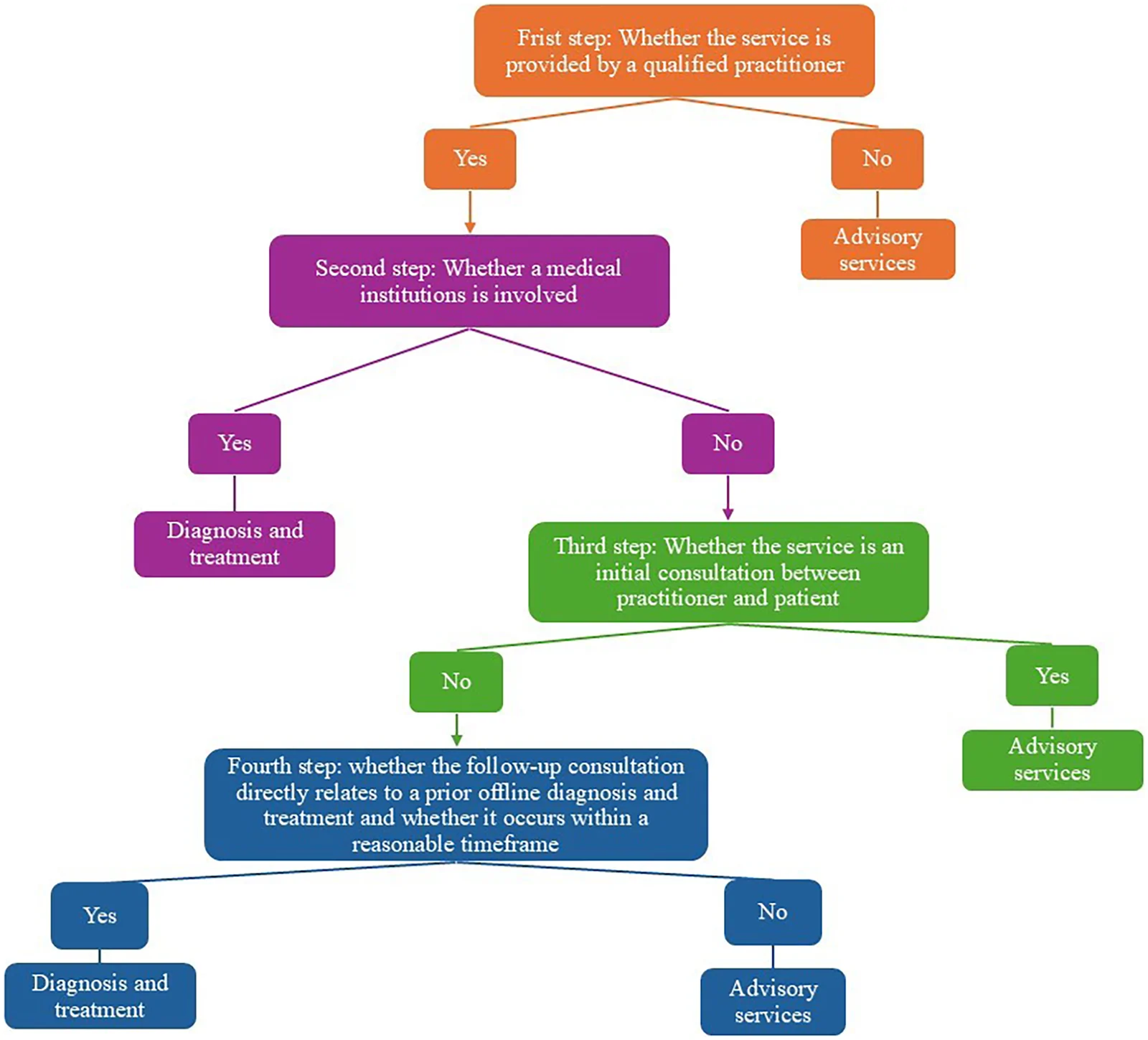
Four-step test for classifying online medical consultation (OMC) services.

If the service is provided by a qualified practitioner, the analysis proceeds to the second step, that is, determining whether a medical institution is involved. If a medical institution is involved—whether a physical facility or an internet hospital—the service should be classified as diagnosis and treatment. The involvement of a licensed medical institution substantially enhances the authority of the service, distinguishing it from health advice offered through mere intermediary platforms. Although current national regulations prohibit initial online diagnosis and treatment, Beijing launched a pilot program permitting initial online diagnosis and treatment for specific pediatric conditions (32). This represents a considerable shift in policy and indicates that online diagnosis and treatment conducted by medical institutions is highly likely to become the norm in the future. Given this trend, OMC services involving medical institutions should be classified as diagnosis and treatment.

If no medical institution is involved—that is, the service is provided through a third-party platform (such as a traditional OMC platform or instant messaging tools) acting merely as an intermediary—the analysis proceeds to the third step. The third step determines whether the service is an initial consultation between practitioner and patient. If it is an initial consultation, the service should be classified as an advisory service. If it is not an initial consultation, the analysis proceeds to the fourth step: assessing whether the follow-up consultation directly relates to a prior offline diagnosis and treatment and whether it occurs within a reasonable timeframe. If both conditions are met—as in the case discussed in Section 2.2.1—the consultation should be classified as diagnosis and treatment. This operational model is particularly common in Beijing, which draws patients nationwide due to its concentration of top-tier medical resources. While many patients travel to Beijing for diagnostic evaluation and initial treatment, requiring them to return in person for every follow-up inquiries is impractical. Consequently, patients frequently maintain contact with their treating doctors through third-party OMC platforms. Conversely, if the follow-up consultation is unrelated to the prior offline care, or if an unreasonable interval has elapsed, the service should be classified as advisory.

### 3.3 Allocating liability according to different operational models

Specific provisions should also be made in the dedicated departmental rule for OMC to address the challenge of determining platform liability. An appropriate approach is to allocate liability based on operational model (Table 2).

For the traditional third-party platform model and the model relying on instant messaging tools, practitioners directly provide consultation services, while platforms serve merely as intermediaries that support communication. This study concurs with the judicial approach adopted by the Beijing Chaoyang Primary People's Court, where practitioners bear liability for medical malpractice,^2^ while platforms incur fault-based liability. Specifically, platforms must fulfill fundamental regulatory obligations and a degree of social responsibility, including verifying practitioner credentials, warning users that services are advisory, maintaining complete and retrievable records, and assisting vulnerable users. For instant messaging platforms, however, it would be unrealistic to require them to verify practitioner credentials or issue such warnings. Their responsibilities should instead be limited to maintaining complete and retrievable records, and assisting vulnerable users.

For operational models involving medical institutions (with or without AI integration), the medical institution should bear liability based on organizational fault, given that it acts as the organizer, designer, and provider of services (33). Furthermore, assigning primary liability to the medical institution would make it easier for the patient to seek redress by removing the need to disentangle joint responsibilities between the institution and the platform (33). If the fault is ultimately attributable to the platform or the AI developer, the medical institution may seek redress from it (33).

For services provided solely by AI, although debate persists over whether property liability should be imposed on AI developers (34), this study argues that the AI developer should bear product liability. This position is supported by several scholars (33, 35). First, AI-powered services meet the statutory definition of a product. Under China's Product Quality Law, a product exhibits two defining attributes: it must be processed and manufactured, and it must be commercialized and circulated (33). The development of multimodal large models involves complex technical processing, making them products of processing and manufacturing (33). These models are subsequently developed by developers in OMC through commercial models, satisfying the requirement of sale and circulation (33). Second, AI applications in healthcare directly impact the right to life and health, where the risk of harm is substantial (36). Applying strict liability under product liability is therefore necessary. As for the platform—given that it does not function exclusively as a healthcare provider—imposing a heightened standard of liability is unwarranted. Instead, the platform should fulfil fundamental regulatory obligations and a degree of social responsibility, including providing clear warnings that services are advisory, exercising prudent oversight, maintaining complete and retrievable records, and offering support to vulnerable users.

## 4 Limitations

This study has several limitations. First, focusing the analysis on Beijing and relying on a limited set of retrieved judicial cases means the identified legal challenges may not be exhaustive. Future research could expand the geographical scope to monitor additional legal issues. Second, the regulation of AI across all its applications—including OMC—remains unresolved and continues to spark considerable debate across academic, legislative, and judicial domains. Moreover, regulatory approaches are rapidly shifting alongside advances in AI technology. While this study offers preliminary recommendations, it does not provide an exhaustive analysis of AI-related questions. Future work could focus specifically on the distinct AI challenges emerging within OMC.

## 5 Conclusion

OMC, a core component of digital health, has undergone rapid development and is expected to continue expanding. However, this growth has been accompanied by legal challenges primarily associated with the nature of OMC services and the difficulties in determining platform liability. The emergence of new operational models and the deeper integration of AI have added further complexity to these issues. Cities such as Beijing, which have distinct advantages in OMC development, experience these challenges more acutely owing to higher adoption rates and more advanced models. Consequently, such cities require a tailored regulatory response. At the center of this response should be a dedicated local administrative measure specifically designed for OMC. The overarching principle is that both issues should be resolved by accounting for different operational models, rather than by applying a single, uniform standard to all cases.

## Data Availability

The original contributions presented in the study are included in the article/Supplementary Material, further inquiries can be directed to the corresponding author.

## Footnotes

^1^This study chose this date because it was the effective date of the two primary regulations governing internet healthcare, namely, the Measures for the Administration of Internet Diagnosis and Treatment (for Trial Implementation), and the Measures for the Administration of Internet Hospitals (for Trial Implementation).

^2^Notably, whether liability attaches, and the specific scope of that liability, depends on whether the service is classified as an advisory service or as diagnosis and treatment.
